# Cemented vs. uncemented reverse total shoulder arthroplasty for the primary treatment of proximal humerus fractures in the elderly—a retrospective case–control study

**DOI:** 10.1186/s12891-022-05994-3

**Published:** 2022-12-01

**Authors:** Manuel Kramer, Martin Olach, Vilijam Zdravkovic, Melanie Manser, Bernhard Jost, Christian Spross

**Affiliations:** Department of Orthopaedics and Traumatology, Kantonsspital St. Gallen, Rorschacherstr. 95, 9007 St. Gallen, Switzerland

**Keywords:** Shoulder, Arthroplasty, Uncemented, Fracture, Reverse, Stress shielding

## Abstract

**Background:**

Uncemented reverse total shoulder arthroplasty (RTSA) for the primary treatment of proximal humerus fractures (PHF) in elderly patients was introduced at our institution in 2017. Recent reports have raised concerns about increased rates of early bone resorption at the proximal humerus with uncemented fracture stems. The aim of this study was to find out whether there was any difference in functional or radiographic outcomes between cemented and uncemented RTSA for PHF.

**Methods:**

Seventeen consecutive patients who underwent uncemented RTSA (group nC) in 2017 and 2018 were age and sex matched (propensity score matching 1:2) to 34 patients with cemented RTSA implanted between 2011 and 2016 (group C) for the primary treatment of PHF. These two groups were compared in terms of clinical and radiographic outcomes at 2 years after the index surgery.

**Results:**

The mean bone quality was low in both groups: in group nC the deltoid tuberosity index (DTI) was 1.43 (1.22–1.72) and in group C 1.42 (1.22–1.67). At the final 2 year follow-up, the relative CS was 98.3% (71–118) in group nC and 97.9% (36–125) in group C (*p* = 0.927); the absolute CS was 70.2 (49–89) in group nC and 68.0 (30–94) in group C (*p* = 0.509). Lucent lines at the humeral site were seen in 8 cases (47%) in group nC and in 13 cases (38%) in group C (*p* = 0.056). Compared to 3% in group C, all patients in group nC showed at least grade 1 and 65% showed grade 3 bone resorption at the proximal humerus (*p* < 0.001).

**Conclusion:**

Compared to cemented RTSA bone resorption at the proximal humerus was significantly more frequent in patients with uncemented RTSA for PHF. So far, this is rather a radiographic than a clinical finding, because both groups showed very satisfying functional outcomes and low revision rates at the 2 year follow-up.

Level of Evidence III.

A retrospective case–control study.

**Supplementary Information:**

The online version contains supplementary material available at 10.1186/s12891-022-05994-3.

## Introduction

With the right indication for the right patient, nearly 2/3 of all proximal humerus fractures (PHF) can be successfully treated conservatively, reserving open reduction internal fixation (ORIF) mainly for high-demand patients with good bone quality [1–3]. For elderly patients with poor bone quality and displaced fractures, primary reverse total shoulder arthroplasty (RTSA) has become a valuable option with reliable and quick return to good shoulder function and to the quality of life they had had before the fracture [1–4].

Uncemented stems for RTSA have been mainly used for indications other than PHF [5]. Despite the risk of decreased primary fixation of the shaft in the metaphyseal area in a PHF, attempts have been made to use uncemented stems also for patients with PHF to avoid cement-specific drawbacks such as longer operation times and difficult revision conditions. At the moment, there are only few data on uncemented RTSA for PHF, some reporting encouraging clinical results [6–11]. However, a few studies also found early bone resorption at the proximal humerus after uncemented RTSA for PHF, a previously rather uncommon radiographic finding [8, 12–16]. Lopiz et al. did not observe negative effects of humeral bone resorption on clinical outcome after two years of follow-up [16]. Since the loss of bone mass could affect possible future revisions, our goal was to develop a better understanding of this phenomenon in RTSA for fracture cases. Thus it was the aim of this study to compare the radiographic and clinical outcomes of cemented vs. uncemented RTSA for PHF in elderly patients with a specific focus on the radiographic analysis for a) signs of bone resorption and early loosening at the humeral site and for b) possible reasons such as implantation technique or humeral loading. We hypothesized that there is no functional difference between cemented and uncemented RTSA in fracture cases after two years of follow-up.

## Patients and methods

A retrospective propensity score matched case–control study of prospectively collected data was performed with data from 2011–2018. From 2011–2016 all RTSA for PHF were cemented (Zimmer anatomical shoulder fracture system: Zimmer, Warsaw, IN, USA). With the introduction of a new shoulder arthroplasty with a more anatomical stem design in 2017 (Medacta shoulder system: Medacta, Castel San Pietro, TI, CH), we started to use uncemented RTSA for PHF. The radiographic and clinical data of all patients with RTSA for PHF are collected prospectively in our institutional registry. The regular follow-up controls are after 3 months and 1, 2, 5 and 10 years post-operatively. The functional outcome is assessed by a specifically trained study-nurse (M.M.) at each follow-up appointment and it includes the absolute and relative Constant score (CS) [17]. Clinical and radiographic complications are evaluated by one of two shoulder specialists (B.J. and C.S.) who, if indicated, also recommend revision surgery.

The data from this registry were used to compare the clinical and radiographic outcomes of patients with RTSA for PHF with uncemented stems (group nC: 2017–2018) to a matched group of patients with cemented stems (group C: 2011–2016). The inclusion criteria were: isolated PHF treated with primary RTSA [3] and a two year follow-up.

**Fig. 1:**
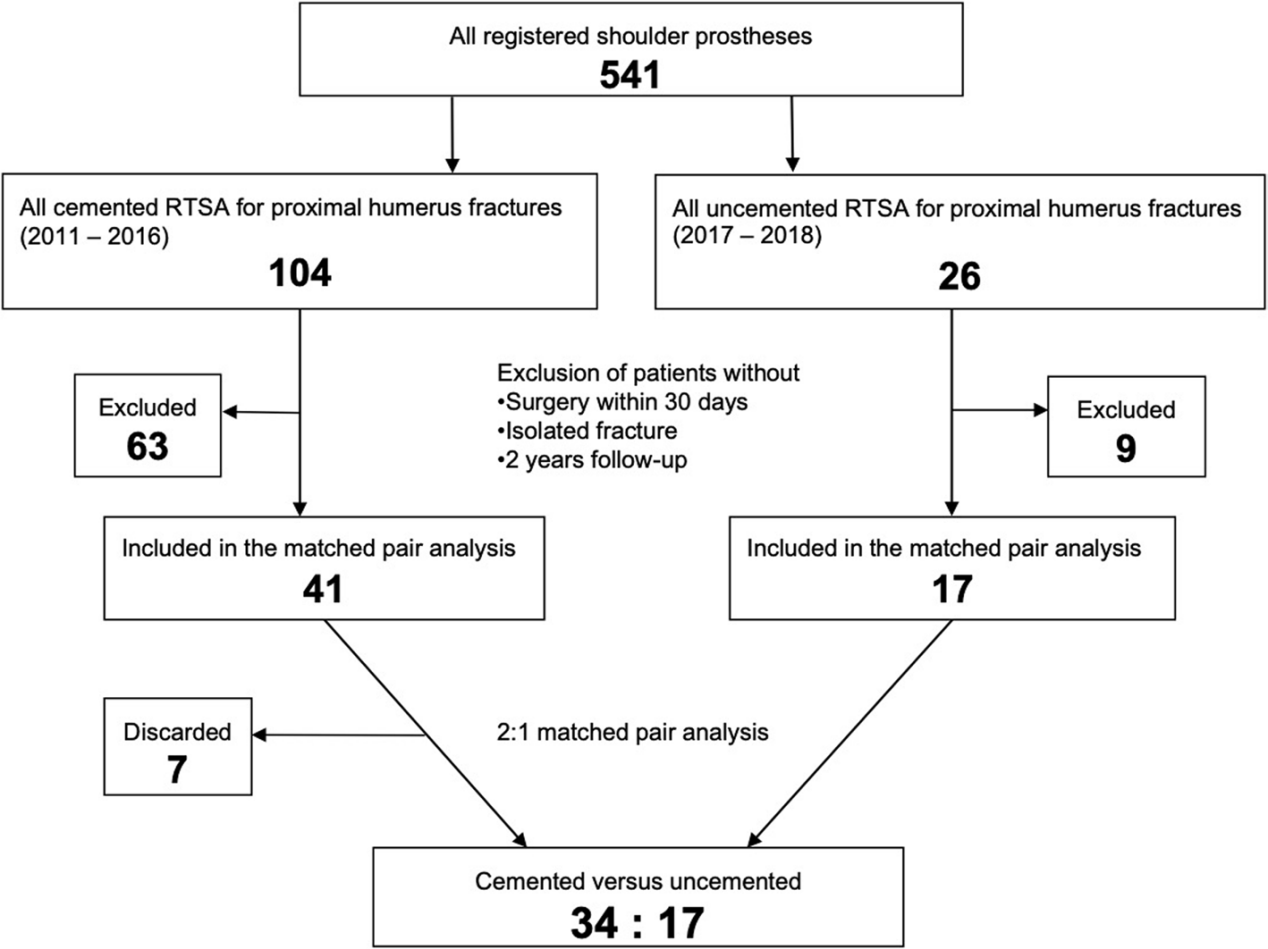
This flowchart shows the patient selection pathway and matching process. Reverse total shoulder arthroplasty (RTSA)

We were able to include 17 consecutive patients treated with uncemented stems to group nC and performed a 1:2 matched pair analysis by age and gender with these 17 patients; for comparison we selected 34 suitable cases from a larger patient collective of the cemented subgroup. The pathway in Fig. 1 shows the patient selection and the matching process.

### Surgical techniques and prosthesis models

All operations were carried out using the delto-pectoral approach in the beach chair position; the same tuberosity refixation technique was used for both cemented and uncemented RTSA following the protocol of Fucentese et al. 2014 [18]. All operations were performed by a total of four orthopedic surgeons, all specialists for shoulder surgery. The Zimmer anatomical shoulder fracture system: (Zimmer, Warsaw, IN, USA) was used for all cemented prostheses using the 3^rd^ generation cementation technique with PALACOS G (Haereus, Hanau, HE, DE). For the uncemented RTSA we used the Medacta shoulder system (Medacta, Castel San Pietro, TI, CH) with standard stem lengths. Postoperative rehabilitation was the same for all patients and included sling immobilization for 6 weeks with pendulum exercises after 2, functional exercises after 4 and weight bearing exercises after 12 weeks.

### Radiographic analysis

All radiographic measurements were performed twice by two of the authors (M.K. and M.O.) The interobserver variability was confirmed by interclass correlation coefficient. For the final radiographic analysis a consensus reading of both raters was used. For the radiological analysis, we reassessed the fractures on CT preoperatively and on X-rays (AP and Neer) pre- and postoperatively, as well as the X-rays of 2 years after RTSA (AP neutral, AP internal rotation, axial, Neer).

We determined bone quality on the AP X-ray of the fracture according to the deltoid tuberosity index (DTI) [19] and the fracture type according to Neer et al. 1970 [20]. The anatomical position and healing of the tuberosity were assessed on the final 2 year post-op X-rays. According to the instructions of Wright et al. 2019 [7], the tuberosity healing was rated as: healed in the anatomic position, dislocated or resorbed.

Loosening of the prosthesis was assessed according to Sperling et al. 2000 [21] based on the number and location of the lucent lines. The occurrence and the grade of scapular notching was evaluated pursuant to the Nerot-Sirveaux Classification [22, 23]. Bone resorption at the proximal humerus was assessed as stated by Aibinder for the grading, and by Denard for the location [12, 15]. Bone resorption was graded from one to three. However the grading was carried out differently on the lateral and medial sides. On the lateral side, grade one is resorption of trabecular bone only, grade two is a thinning of the cortex and grade three is a complete cortical resorption down to the prosthesis. On the medial side, bone resorption was only registered when the cortex was thinned and graded from one to three according to the extent of the resorption zone. If the resorption occurred within the first third of the ingrowths surface, it was classified as grade one. If resorption happened from the second to the last third it was designated as grade two and absorption beyond the ingrowth surface was grade three [12]. The stem to humerus filling ratios were measured for all uncemented prostheses following the instructions of Denard et al. 2018 [15].

### Statistical analysis

All statistical analyses were performed using R (R: A language and environment for statistical computing: R Foundation for Statistical Computing, Vienna, Austria—URL http://www.R-project.org/). We applied R MatchIt package for propensity score matching at a 1:2 ratio. Descriptive statistics included means, ranges, standard deviations and proportions. To assess the interobserver variability we calculated the interclass correlation coefficient ICC3 according to Shrout et al. 1979 [24]. Comparative statistics included t-test and Chi-square test (Wilcoxon and Fisher exact test were applied where alternatively appropriate). The confidence level for rejecting the null hypothesis was set at 95% (*p*-value < 0.05).

## Results

### Demographics and functional outcomes

A total of 51 reverse shoulder arthroplasties as primary therapy for PHF were analyzed; their general data are depicted in Table 1. As expected, both groups had similarly poor bone quality as measured by the DTI (*p* = 0.842). The interobserver reliability of the DTI measurements was high between the two examiners with an ICC of 0.85 (ICC3 according to Shrout; *p* < 0.001).

**Table 1:** Comparison of demographic data, bone quality and fracture configuration between cemented and uncemented groups

	**All (*n* = 51)**				**Cemented (*n* = 34)**				**Uncemented (*n* = 17)**				
	Mean	SD	min	max	Mean	SD	min	max	Mean	SD	min	max	*p*-value
**Age (years)**	74	6	58	90	74.4	5.4	64	84	74.1	7.8	58	90	0.903
**DTI**	1.42	0.13	1.22	1.72	1.42	0.12	1.22	1.67	1.43	0.15	1.22	1.72	0.842
	**N**	**%**			**N**	**%**			**N**	**%**			
**Sex (m/f)**													0.057
male	8	16			3	9			5	29			
female	43	84			31	91			12	71			
**Laterality (r/l)**													0.839
right	20	39			13	38			7	41			
left	31	61			21	62			10	59			
**Fracture type**													0.822
1-part	0	0			0	0			0	0			
2-part	4	8			3	9			1	6			
3-part	30	59			19	56			11	65			
4-part	17	33			12	35			5	29			

*DTI* Deltoid tuberosity index, *rel CS* Relative Constant score

After two years excellent functional results were found in both groups. Mean follow up was 27 months (± 9.38). The relative CS was 97.88% in group C and 98.29% in group nC (*p* = 0.927); the absolute CS was 68.00 in group C and 70.24 in group nC (*p* = 0.509).

### Radiographic outcomes

All radiographic comparisons were made on the two year follow-up X-rays, as listed in Table 2. There was low occurrence of scapular notching in both groups (6% in group nC and 18% in group C). The greater tuberosity healed in the anatomical position for 71% in group nC and 79% in group C, while the values for anatomical healing of the lesser tuberosity were slightly higher at 82% in group nC and 94% in group C. In both groups no sign of loosening was seen at the glenoid. We found 3 or more lucent lines at the humeral component in 18% of the cemented group and in 6% of the uncemented group. None of these was considered to be “at risk for loosening” (width > 2 mm) [21].

**Table 2:** Comparison of the radiographic analysis between cemented and uncemented groups.

	**Cemented group (*n* = 34)**		**Uncemented group (*n* = 17)**		
	N	%	N	%	*p*-value
**Bone resorption**					< 0.001
yes	3	9	17	100	
no	31	91	0	0	
**Bone resorption grade medial**					< 0.001
0	34	100	6	35	
1	0	0	0	0	
2	0	0	10	59	
3	0	0	1	6	
**Bone resorption grade lateral**					< 0.001
0	31	91	1	6	
1	0	0	0	0	
2	2	6	5	29	
3	1	3	11	65	
**GT healed**					0.48
yes	27	79	12	71	
no	7	21	5	29	
**LT healed**					0.18
yes	32	94	14	82	
no	2	6	3	18	
**Scapular notching**					0.24
yes	6	18	1	6	
no	28	82	16	94	
**Scapular notching grade (1–4)**					0.18
0	28	82	16	94	
1	5	15	1	6	
2	1	3	0	0	
> 2	0	0	0	0	
**Lucent zones**					0.06
0	21	62	9	53	
1–2	7	21	7	41	
3–7	6	17	1	6	

*GT healed* Greater tuberosity healed in anatomical position, *LT healed* Lesser tuberosity healed in anatomical position

**Fig. 2:**
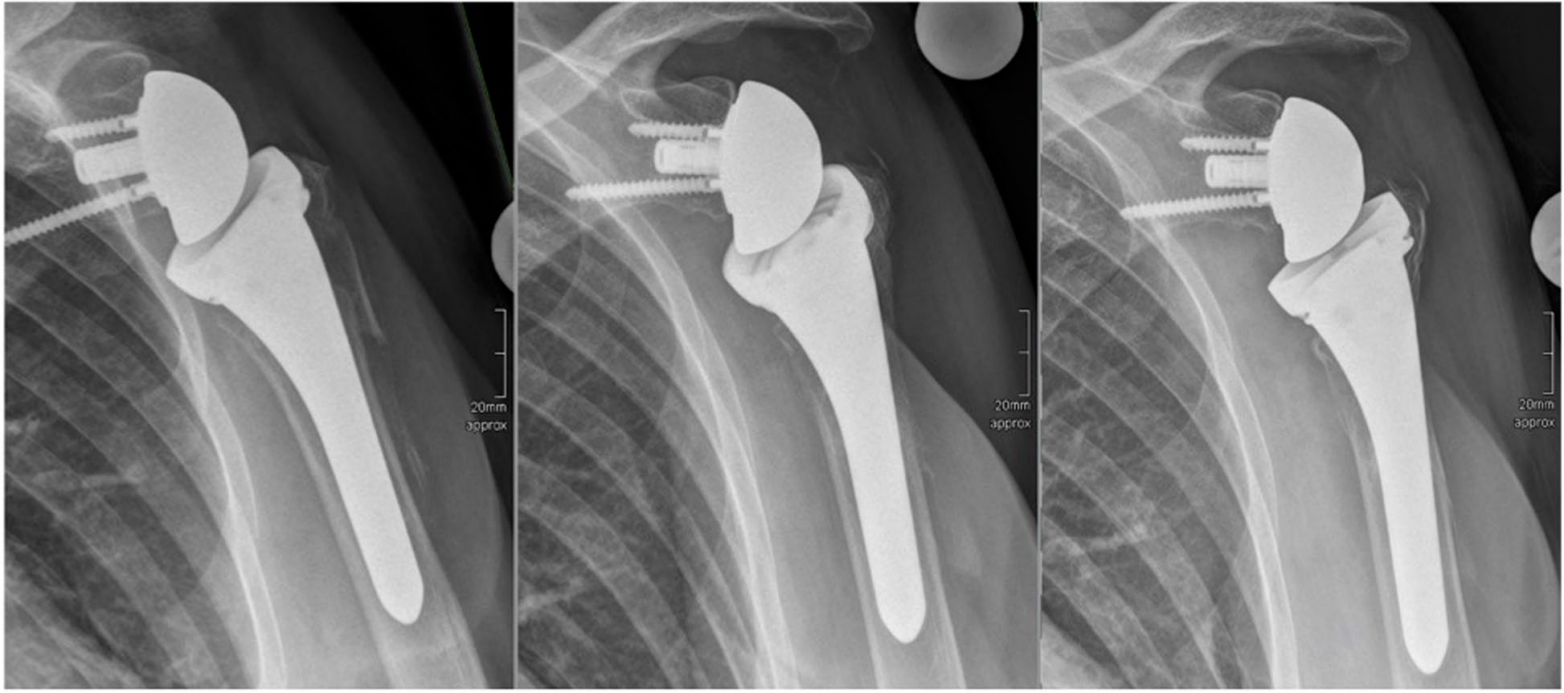
This example shows the typical progression of bone resorption effects on the lateral side of the humeral stem. You can see a good reduction of all bone fragments at 3 months after implantation (left), bone resorption effect grade 2 after one year (middle) and grade 3 after two years (right). A similar process, but less pronounced, can also be seen at the medial side

In group nC all patients showed bone resorption of at least level 1 compared to only 9% in group C (*p* < 0.001). Bone resorption was more frequent on the lateral than on the medial side of the proximal humerus. An example of the process of lateral bone resorption over time is shown in Fig. 2. Distal stem/humerus filling ratios (dFR) of all uncemented prostheses (*n* = 17) were measured; there was no difference between the cases with severe bone resorption [(*n* = 11) (dFR = 0.65)] and the other cases [(*n* = 6) (dFR = 0.62)].

### Complications and revisions

One patient in group nC needed several surgical revision interventions with temporary removal of the prosthesis because of implant infection. Two patients in group C needed revision surgery. One because of malpositioning of the glenoid component during the primary procedure. The revision could be performed during the same hospital stay. The other one due to a periprosthetic fracture nearly two years after surgery, which we were able to stabilize using cerclages and plating.

## Discussion

This study presents one of the few comparative series of patients with uncemented and cemented stems for primary fracture RTSA. It includes a specific analysis of bone resorption at the proximal humerus with special regard to early shaft loosening and functional outcome. Within a follow-up period of two years we found consistently good functional results in both the uncemented (group nC) and the cemented control group (group C). Revision rates were generally low and importantly, no revision due to (early) shaft loosening was necessary. These data also correspond to the currently available literature on this topic [8, 10, 11, 16]. The radiographic outcome was comparable in both groups too except for the significantly higher incidence of bone resorption at the proximal humerus in the uncemented group. In a retrospective study published in 2022, Lopiz et al. found significantly more stress shielding in uncemented RTSA in fracture cases [16]. Consistent with our results they could not find any correlation between humeral stem loading and stress shielding, but discussed reduced bone quality as possible risk factor. Our quantitative measurements of bone quality with DTI could not confirm their assumption. However, our study was underpowered for this subgroup analysis. To reduce the bias due to the expected poorer bone quality in elderly people, we prospectively collected data and performed age and gender matched pair analyses.

Bone resorption at the proximal humerus in uncemented RTSA was first described in patients with PHF in 2019 [8]. Two years earlier we started with uncemented prostheses using a different implant and also found this radiographic phenomenon in our follow-up investigations. The stress shielding effect is mainly known from hip arthroplasty where the greater trochanteric region may show bone resorption as it is shielded from stress by the distal bone loading of e.g. an extensively porous coated stem [25]. Other known risk factors are: large-diameter stems, Co-Cr alloy stems and round, cylindrical stem designs. At the shoulder the stress shielding effect must be different from the hip as the above-mentioned risk factors could not, to a large extent, be confirmed in this study. The uncemented implant used in our study is coated proximally (thus proximal bone loading) and has a titanium calcar-shaped shaft design. However, the filling ratio of the prosthesis compared to the bone has been described as a potential risk factor for proximal humerus bone loss in patients with an anatomical shoulder arthroplasty [15]. This may be one possible explanation for the high percentage of stress shielding after RTSA for PHF. Compared to an intact proximal humerus, the shaft size chosen in a fracture situation may be slightly bigger aiming for more distal metaphyseal primary fixation, as the metaphyseal area is usually also fractured, at least proximally. However, we did not find a higher distal filling ratio with severe stress shielding in the uncemented subgroup. Furthermore, bone resorption may be more obvious in PHF as the fractured metaphysis and tuberosities may not heal completely back onto the shaft resulting in bone loss in this area as well. However, this would be the same for cemented and uncemented stems. It may thus not be surprising that we found more stress shielding in our uncemented fracture group patients (100%), compared to other studies with uncemented hemiprostheses and anatomical total prostheses (63%) [26] or uncemented RTSA (68%) [14] for degenerative cases.

### Limitations

Even though our data were collected prospectively, this current analysis is retrospective with a relatively small number of consecutive cases of primary uncemented RTSA for PHF. This is comparable to the available literature; however it is still underpowered for certain subanalyses concerning the effects of bone quality and shaft size on stress shielding after uncemented stems.

The use of two different implants in our groups may be a drawback as well. A direct comparison of the cemented vs. uncemented stems may be biased by the different designs of these prostheses.

Two years of follow-up are certainly not enough to draw definitive conclusions whether or not uncemented stems are safe in the long term. The relevance of this stress shielding for revision surgeries needs to be further investigated in the future.

## Conclusion

Uncemented and cemented reverse total shoulder arthroplasty for PHF in elderly osteoporotic patients lead to excellent functional results with low complications and revision rates after 2 years. Our study revealed that the incidence of bone resorption is higher in uncemented RTSA; this is, however, so far without clinical relevance. This radiographic finding at the proximal humerus is not yet fully understood, and a longer follow-up will be needed to better understand its relevance for long-term outcomes.

## Supplementary Information

**Additional file 1.**

## Data Availability

The datasets used and/or analysed during the current study are available from the corresponding author on reasonable request.

## Abbreviations

RTSA: Reverse total shoulder arthroplasty
PHF: Proximal humerus fracture
group C: Group with cemented stems
group nC: Group with uncemented stems
ORIF: Open reduction internal fixation
CS: Constant score
