# Supplementary material for: Cemented vs. uncemented reverse total shoulder arthroplasty for the primary treatment of proximal humerus fractures in the elderly—a retrospective case–control study
Source: BMC Musculoskelet Disord. 2022 Dec 1;23:1043. doi: 10.1186/s12891-022-05994-3 (PMC9714093; doi:10.1186/s12891-022-05994-3)
Supplement: Supplementary file 1 — Additional file 1. [file 12891_2022_5994_MOESM1_ESM.docx]

Supplementary Material 1


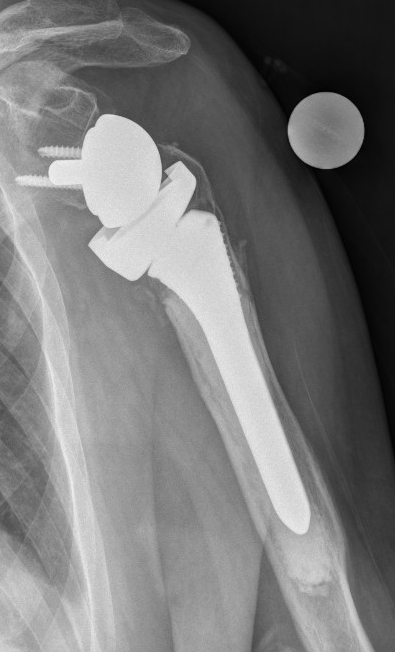

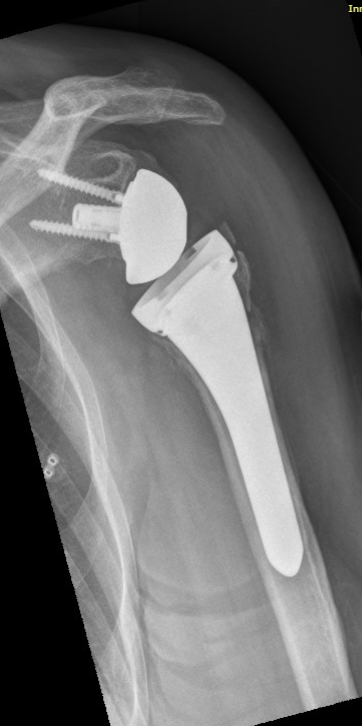
This picture shows the two prosthesis models examined 3 months postoperative. On the left side you can see the prosthesis from Medacta (Medacta shoulder system (Medacta, Castel San Pietro, TI, CH) ) which was implanted cementless. On the right side you can see the prosthesis from Zimmer (Zimmer anatomical shoulder fracture system (Zimmer, Warsaw, IN, USA)) which was used with a cemented stem.
